# Application of the target trial emulation framework to external comparator studies

**DOI:** 10.3389/fdsfr.2024.1380568

**Published:** 2024-04-11

**Authors:** Kellyn Arnold, Luis Antunes, Briana Coles, Hopin Lee

**Affiliations:** ^1^ IQVIA, Methods and Evidence Generation, London, United Kingdom; ^2^ IQVIA, Methods and Evidence Generation, Lisbon, Portugal

**Keywords:** external comparator, external control, target trial emulation, causal inference, comparative effectiveness, health technology assessment

## Abstract

External comparator (EC) studies are increasingly being used to generate evidence that supports the evaluation of emerging pharmacological treatments for regulatory and health technology assessment (HTA) purposes. However, the reliability of evidence generated from EC studies can vary. In this paper, we outline how an existing framework for causal inference, the target trial emulation (TTE) framework, can be appropriately applied to improve the design and analysis of EC studies. Applying the TTE framework involves specifying the protocol of an ideal target trial which would answer the causal question of interest, then emulating its key elements under real-world (RW) settings. We describe each component of the original TTE framework and explain how it can be applied to EC studies, supplemented with practical recommendations. We also highlight special considerations and limitations in applying the TTE framework to EC studies. We describe how the TTE framework can be applied to improve the clarity, transparency, and reliability of evidence generated from EC studies.

## 1 Introduction

Randomised controlled trials (RCTs) are considered the gold standard for answering causal questions about the comparative efficacy or safety of health-related interventions (European Medicines Agency). However, there are many causal questions for which RCTs may not be ethical, feasible, or timely. In such cases, observational real-world data (RWD) such as disease registries, hospital or pharmacy claims, or electronic health records (EHRs) can be used to answer causal questions if key sources of biases can be adequately addressed (Hernan and Robins, 2020).

RWD are increasingly being used in submissions to regulatory bodies such as the UK Medicines and Healthcare products Regulatory Agency (MHRA), the European Medicines Agency (EMA), and the US Food and Drug Administration (FDA) (European Medicines Agency, 2023; U.S. Food and Drug Administration, 2018; Cave et al., 2019). RWD are also used to generate evidence for health technology assessment (HTA) bodies such as the UK National Institute for Health and Care Excellence (NICE), German Gemeinsamer Bundesausschuss (GBA), and French Haute Autorité de Santé (HAS), for evaluating the economic impact and value of medicinal products (National Institute for Health and Care Excellence, 2022; Curtis et al., 2023).

With the increasing availability and quality of RWD in recent times, RWD are also being used to create a comparator arm to a ‘referent’ single-arm trial (SAT) or the active arm of a two-arm parallel RCT. Studies that make use of these designs are typically labelled external comparator (or control) (EC) studies, and they have been used to generate evidence that supports the evaluation of emerging pharmacological treatments for regulatory and HTA purposes (Seo, 2023). Although the concept of external controls was described in published literature as early as 1976, EC studies have received renewed attention within the drug development community over the last several years (Pocock, 1976). A recent systematic review identified 64 regulatory and 70 HTA submissions between January 2015 and August 2021 which included primary evidence from a SAT supplemented by an EC derived from RWD, for which the most common therapeutic areas were oncology, haematology, neurology (Sola-Morales et al., 2023).

A key benefit of the EC design is that it allows for broader contextualisation of pivotal trial results with reference to alternative treatments in RW settings (Carrigan et al., 2020; Sola-Morales et al., 2023). However, the strength of evidence generated from EC studies is often variable, and regulatory and HTA review committees have highlighted several biases as important limitations to the evidence generated from these studies (Jaksa et al., 2022; Sola-Morales et al., 2023). In light of this, regulatory and HTA bodies have released general guidance documents aimed at improving the quality of EC studies (National Institute for Health and Care Excellence, 2022; U.S. Food and Drug Administration, 2023). However, these documents do not cover how existing frameworks for causal inference, such as the target trial emulation (TTE) framework, can be appropriately applied to EC studies (Hernán and Robins, 2016). In this paper, we outline how the TTE framework can be applied to improve the robustness of EC studies.

## 2 Application of the target trial emulation framework to external comparator studies

The idea of emulating randomised experiments using observational data has been documented since the 1950s (Cox, 1958; Rubin, 1974). In 2016, Hernan and Robins formalised this idea as the TTE framework by outlining a template for emulating hypothetical target trials with observational data (Hernán and Robins, 2016). Since then, the TTE framework has been extensively used to design numerous comparative effectiveness and safety studies for generating real-world evidence (García-Albéniz et al., 2017; Caniglia et al., 2018; Caniglia et al., 2020; Emilsson et al., 2023).

Applying the TTE framework first involves specifying the protocol of an ideal, hypothetical ‘target’ trial which would answer the causal question of interest. Key elements of the target trial—including the eligibility criteria, treatment strategies, treatment assignment procedures, assignment of time zero, follow-up period, outcomes, causal contrasts and estimands, and analysis plan—are then emulated using RWD. Several published studies have demonstrated how applying the TTE framework can successfully reduce the impact of important biases and promote transparency in observational analyses (Hernán et al., 2016; García-Albéniz et al., 2017; Bakker et al., 2021).

It may appear that the TTE framework can be directly applied to the EC setting by conceptualising EC studies as typical observational causal analyses. However, there are methodological nuances in the application of the TTE framework to EC studies due to the combination of data from two distinct settings (i.e., experimental and observational) that do not share a common data-generating mechanism.

In the following sections, we describe each component of the original TTE framework and explain how it can be applied to EC studies, supplemented with practical recommendations. We also highlight special considerations and limitations in applying the TTE framework to EC studies. Throughout, we assume that appropriate data source(s) have been selected using existing tools such as the revised Structured Process to Identify Fit-for-Purpose Data (SPIFD2) framework, and that the data are relevant to target stakeholders in terms of clinical relevance and geographical coverage (Gatto et al., 2023). Note that SPIFD2 framework explicitly highlights the utility of considering the required elements of a hypothetical target trial when assessing data source(s), thereby allowing for an iterative approach to study design and data source selection for EC studies (Gatto et al., 2023).

### 2.1 Eligibility criteria

The TTE framework emphasises that an important aspect of emulating a target trial involves defining a target population. The target population can be viewed as the population the investigator and stakeholder wish to make inferences about. This target population is often defined using a set of eligibility criteria that are broad enough to enrol a sufficient number of patients, but specific enough to identify a population that could potentially benefit from the drug being investigated (European Medicines Agency, 2020; Hornberger and Rangu, 2020).

In the EC setting, the eligibility criteria are specified in the referent trial protocol. Therefore, in principle, identical criteria would be used to identify a group of RW patients who would be deemed eligible for the referent trial. In practice, not all eligibility criteria from the referent trial can be applied due to RWD limitations. In such cases, proxy measures may be used to emulate certain criteria, and some may need to be omitted—which can lead to imperfect specification of the target population. The impact of omitting any criteria in an EC study must be carefully assessed on a case-by-case basis. For instance, eligibility criteria which aim to cover the ethical or practical requirements of subjecting patients to experimental conditions may seem irrelevant to RWD, but their exclusion may introduce bias. While the exclusion of non-English speakers in a trial recruiting from an English-speaking country may seem irrelevant, including such patients in the EC may introduce cultural and ethnic diversity which could compromise their exchangeability with patients from the referent trial. Decisions about which criteria to emulate may also be informed by practical considerations around sample size, since the application of each individual criterion is likely to increase exchangeability at the cost of reducing the sample size available for analysis. Sensitivity analyses may be performed to assess the impact of applying or omitting certain eligibility criteria on study results.

In some cases, omitting an eligibility criterion (e.g., due to RWD limitations) may lead to violations of the positivity assumption, which is required for causal inference (Zhu et al., 2021). For example, if the active drug being investigated in the referent trial is contraindicated in patients with photosensitivity, ignoring this criterion for the EC (e.g., to preserve sample size or because photosensitivity is not well recorded) would lead to a situation in which photosensitive patients who should not receive the intervention would be included in the EC. Ultimately, after all selected (and perhaps adapted) eligibility criteria from the referent trial are applied, all eligible patients in the EC should have a non-zero probability of receiving the active treatment to meet the positivity assumption.

### 2.2 Treatment strategies

A target trial should specify at least two treatment strategies, one involving the active agent under investigation (i.e., the treatment of interest) and another distinct treatment strategy (i.e., the comparator). Common comparators include no treatment, routine treatment (or treatment as usual), standard-of-care (SoC) or ‘best practice’, or a particular active agent (Freedland et al., 2019; Nair, 2019). The choice of comparator depends on the purpose of the study—that is, whether the purpose is to evaluate if the treatment of interest works at all or to determine how well it works relative to current practice or another drug (Freedland et al., 2019).

In the EC setting, treatment strategies must be well-defined and relevant to clinical and policy decision making (Rippin, 2024). The description of a treatment strategy for the comparator should be made as unambiguous as possible to avoid violating the causal consistency assumption, which requires that all versions of a treatment strategy with respect to a specific dose, frequency, duration, and route of administration would have the same effect (Cole and Frangakis, 2009). The treatment strategy should also specify discontinuation rules and concomitant medications that can or cannot be taken. This can be relatively straightforward when a treatment strategy involves point intervention for a single treatment; however, for sustained treatment strategies or a heterogeneous mix of SoC treatments, specification of the treatment strategy can be more complex. Moreover, in situations where SoC treatments have improved over time, the use of historical RWD may result in a biased comparison between treatment arms. As with the application of eligibility criteria, the definition of a comparator treatment strategy therefore requires balancing sample size considerations with internal validity. Although a broad definition of SoC is likely to increase the number of patients, it is also likely to increase the chances of confounding. Moreover, the results may have less external validity if SoC differs across jurisdictions or changes over time.

### 2.3 Treatment assignment procedures

In an ideal target trial, patients would be randomly assigned to different treatment strategies to ensure that the groups of patients allocated to each of the treatment strategies have a similar distribution of baseline characteristics and are therefore ‘exchangeable’. In other words, randomisation ensures that the expected future outcomes of each treatment group would be equivalent, on average, in the absence of treatment (Hernán and Robins, 2006). Emulation of random assignment in a typical TTE analysis would involve identifying and adjusting for a set of variables which are likely to confound the relationship between the treatment and outcome. The goal of this approach is to achieve *conditional* exchangeability between the treatment groups at the time of treatment initiation and throughout follow-up (if time-varying confounders are used for adjustment) (Hernán and Robins, 2016).

In the EC setting, conditional exchangeability can be achieved by identifying and adjusting for all treatment effect modifiers and confounders (Dong et al., 2020). Potential confounding factors should be identified using directed acyclic graphs (DAGs), which distil expert clinical knowledge and theory into easily interpretable diagrams and produce a minimum but sufficient set of covariates for adjustment (Rodrigues et al., 2022). However, if the referent trial and/or the chosen source(s) of RWD do not contain sufficient information on likely confounders, successful emulation of random assignment will not be possible and residual confounding will remain (Hernán and Robins, 2016). In these situations, it is also valid to question whether the RWD source is still fit-for-purpose if important covariates are not available. If so, simulation-based analyses may be performed to assess the sensitivity of results to known or suspected confounders, or indirect approaches (e.g., negative controls or instrumental variables) may be used to assess sensitivity to unmeasured confounding (Lin et al., 1998; Martens et al., 2006; Lipsitch et al., 2010; Kutcher et al., 2021).

### 2.4 Time zero

‘Time zero’ refers to the point at which patient follow-up begins; it is also commonly referred to as ‘baseline’ or ‘index (date)’. In an RCT, time zero is naturally defined as the timepoint at which patients are randomised, initiate treatment, and begin to be observed for any outcome event(s) of interest. The alignment of these timepoints ensures that selection and immortal time biases are prevented (Hernán et al., 2016).

In the EC setting, time zero should be aligned across all treatment arms. For an EC with an active comparator treatment, the assignment of time zero is generally straightforward: time zero begins at treatment initiation for all patients in the referent trial and the comparator arm. However, when patients in the comparator arm can meet eligibility criteria at multiple time points (e.g., because the comparator treatment strategy is ‘no treatment’ or is an active treatment that can be delivered in multiple treatment lines), there are a variety of approaches to defining time zero (Hernán and Robins, 2016; Kutcher et al., 2021; Hatswell et al., 2022). For specific situations, randomly assigning an eligible timepoint to patients in the EC arm, matching pre-treatment person-time between the EC and referent trial arms, and defining multiple time zeros across the study period by creating a series of nested trials have shown to be valid approaches to assigning index dates to patients in the EC arm. For a comprehensive evaluation of various approaches to selecting time zero in EC studies, refer to Hatswell et al. (Hatswell et al., 2022).

### 2.5 Follow-up period

In an RCT, the follow-up period refers to the time during which patients are observed. Follow-up begins at time zero and ends at a specific timepoint as defined by the protocol. For time-to-event analyses, patients may experience the event of interest or be censored when they are lost to follow-up or the study period ends (i.e., administrative censoring). Patients experiencing competing events (e.g., death) may also be censored for some analyses, and handling such events often require the application of complex methods as determined by the targeted estimand (Young et al., 2020).

Under the TTE framework, a fixed follow-up period and pre-specified conditions for censoring patients should be defined (Hernán and Robins, 2016). In the EC setting, both definitions should be harmonised across the referent trial and EC, which typically involves applying the specified follow-up period and definitions of censoring events from the referent trial to the EC. However, data limitations may require modifications to be made to the referent trial instead. For instance, the maximum follow-up time may need to be limited by the minimum duration of follow-up available in the referent trial and the EC.

Biases arising from informative censoring are likely to be exacerbated in EC studies. Although RCTs typically attempt to minimise losses to follow-up through strict monitoring, such attempts are not typically made in RWD. Thus, differential censoring mechanisms can be expected in EC studies, for example, if patients in an EC arm have more comorbidities and experience competing events earlier than patients in the referent trial. This kind of informative censoring can lead to post-baseline selection bias, since patients who remain in the study may not be representative of those who are censored (Kutcher et al., 2021). Where appropriate, statistical approaches such as inverse probability of censoring weighting should be applied to correct for informative censoring (Robins et al., 2000; Fewell et al., 2004; Seaman and White, 2013; Willems et al., 2018).

### 2.6 Outcomes

RCTs are required to pre-specify a set of outcomes or endpoints that will be evaluated during the follow-up period, including how such outcomes will be measured. The choice of outcomes will depend on the study objectives and the target audience. For example, trials intended for regulatory submission often include efficacy outcomes (e.g., overall survival) and safety event outcomes (e.g., number of serious adverse events), whereas trials intended for HTA bodies may include other outcomes such as duration of response and time to next treatment (Delgado and Guddati, 2021). In some trials, patient-reported outcomes (PROs) such as quality of life will also be used, often as secondary outcomes evaluated at fixed time points. To mitigate potential ascertainment or detection biases in outcome evaluation, outcomes may be adjudicated by independent trial administrators who are blinded to patients’ treatment allocation (Mansournia et al., 2017).

In the EC setting, the referent trial protocol can be leveraged to define a set of pre-specified outcomes. In principle, the same outcomes (or a subset thereof) will be evaluated in the EC according to the same conditions, such that the timing, duration, and method of measurement are consistent across the referent trial and EC. However, in practice, there are likely to be several challenges to this approach. For instance, it is unlikely that all pre-specified outcomes from the referent trial can be ascertained from RWD. For example, oncology outcomes that rely on tumour response criteria are challenging to apply and are rarely available in RWD. For such outcomes, it may be necessary to use proxy measures or alternative methods of ascertainment. Moreover, outcomes that are collected in the RW may have been measured under different conditions to those in the trial setting, and there may be a greater degree of underreporting of certain RW outcomes (e.g., depending on severity or setting of care). It is also not possible to emulate blinded outcome assessment in an EC study, since physicians in a RW setting will be aware of the treatment a patient is receiving. Thus, one should assess how well the outcomes from the referent trial can be emulated in the EC and prioritise those which are least likely to induce misclassification or detection bias but are still relevant to the study objectives. Where possible, validated code lists should be used to ascertain the outcomes of interest. Re-adjudication of RW outcomes by an independent blinded reviewer or panel may also be possible in some situations, but this approach can be resource intensive (U.S. Food and Drug Administration, 2023). When outcome misclassification is suspected, sensitivity analyses such as quantitative bias analysis may also be used (Fox et al., 2023).

### 2.7 Causal contrasts and estimands

In an RCT, there are two common causal estimands of interest: intention-to-treat (ITT) and per-protocol (PP) (Murray et al., 2021). The ITT captures the average causal effect of treatment *allocation*, regardless of whether patients initiate or adhere to their assigned treatment strategy. When patients in an RCT do not fully adhere to their assigned treatment strategies or are differentially lost to follow-up due to treatment, the ITT is unlikely to reflect the true effect of the specified treatment strategy. In such cases, the PP effect may also be estimated, as it represents the average causal effect of treatment had all patients fully adhered to their assigned treatment strategy as specified by the trial protocol. If all patients perfectly adhere to their assigned treatment strategy and are not differentially lost to follow-up, the ITT and PP estimands will be equivalent. Historically, the ITT has been preferred over the PP because it preserves exchangeability of the treatment groups at baseline due to randomisation. In contrast, the PP is subject to post-baseline confounding since deviations from an assigned treatment strategy during follow-up are unlikely to be random; consequently, methods which account for post-baseline events are required (Seaman and White, 2013; Murray et al., 2021).

In EC studies, both the ITT and PP estimands can be estimated with adaptations. The standard ITT definition is difficult to apply in the EC setting because we typically observe the initiation of a treatment strategy rather than assignment (i.e., randomisation) in the EC arm. Hence, the ITT in EC settings could be more appropriately considered the effect of treatment initiation rather than treatment allocation. The PP effect in an EC study is analogous to the PP effect in an RCT and accordingly requires a detailed description of the comparator treatment strategy (see section 2.2) and the use of the appropriate methods for baseline and post-baseline confounding adjustment (Toh et al., 2010; Hernán and Hernández-Díaz, 2012; Maringe et al., 2020). For more detail on estimands in EC studies, refer to Rippin (Rippin, 2024).

Another consideration is that the EC study sample represents a case-mix of at least two populations with different selection mechanisms (i.e., referent trial and RW). Therefore, the average treatment effect may differ across the following target populations: treated population (i.e., represented by the referent trial cohort), untreated population (i.e., represented by comparator treatment cohort), overall population (i.e., represented by combined treated and comparator cohorts), and overlap population (i.e., represented by the overlapping population between the treated and comparator cohorts) (Greifer and Stuart, 2021). The nomenclature of estimands that refers to these target populations are often termed the average treatment effect in the treated (ATT), average treatment effect in the control (ATC) or untreated (ATU), the average treatment effect (ATE), and the average treatment effect in the overlap population (ATO), respectively. In an EC setting, the ATE represents a population that comprises a combined subset of patients that are represented by the trial and RW EC, and thus may not translate to a realistic target population that is of relevant interest to regulators or HTA bodies. In most cases, the ATT and/or ATC would be considered the primary estimand of interest for EC studies.

### 2.8 Statistical analysis

RCTs seek to minimise sources of bias and the potential for spurious findings primarily through randomization and study design and secondarily through careful application of statistical methods (European Medicines Agency, 2020; International Council for Harmonisation of Technical Requirements for Registration of Pharmaceuticals for Human Use, 2022). To minimise the potential for data-driven findings, all primary, secondary, and/or exploratory analyses are pre-specified. Other procedures which require pre-specification include defining how missing data will be handled, which statistical model(s) will be used, what data transformations will be applied, what factors will be adjusted for and how, and what adjustments to the level of significance will be made when multiple primary analyses or treatment comparisons are planned (European Medicines Agency, 2020). In order to improve the precision of the estimated effect(s), analyses are typically adjusted for a number of baseline covariates, including any factors used in the stratified randomisation procedure and other clinically relevant factors known *a priori* to be strongly associated with the outcome (Committee for Medicinal Products for Human Use, 2015). Exploratory analyses may also be performed to evaluate variations in the causal effect across different subgroups (European Medicines Agency, 2020). Where the PP effect estimand is of interest, the treatment strategy must be clearly defined and adjustment methods for post-baseline events are required (e.g., through inverse probability of censoring weights) (Toh et al., 2010; Hernán and Hernández-Díaz, 2012; Seaman and White, 2013; Murray et al., 2021). The validity of modelling assumptions should be explored through model diagnostics and sensitivity analyses.

Many of the above analytical considerations are required for an EC study; however, there are a few key differences. For instance, the absence of random treatment assignment in the EC setting means that exchangeability of treatment groups is not granted by design. Covariate adjustment for potential treatment effect modifiers and confounding factors is therefore needed to reduce bias and achieve conditional exchangeability (Dong et al., 2020). Minimising baseline differences between the referent trial and EC treatment arms is typically achieved through propensity score (PS) methods (e.g., weighting or matching) (Ross et al., 2015). In general, weighting is preferred to matching as it does not discard patients from the analysis, although caution is needed when extreme PSs are observed. PS diagnostics are always recommended to evaluate if covariate balance between treatment groups has been achieved following the weighting or matching procedure. The target population for the analysis should also inform the method used, as explained in detail by Greifer and Stuart (Greifer and Stuart, 2021). Depending on the target audience of the EC study, additional sensitivity analyses may also be required to evaluate the robustness of study results to data curation, study design, residual confounding, and analytical decisions (National Institute for Health and Care Excellence, 2022).

Another key consideration for EC studies is the occurrence of missing baseline information, which is crucial for successfully conducting bias adjustments of the treatment effect (e.g., by PS methods) and for defining the target population according to the eligibility criteria. As such, the handling of missing data should be a major focus of the protocol and the statistical analysis plan. Various methods for handling missing data may be used, including multiple imputation, mixed models, and generalised estimating equations (Committee for Medicinal Products for Human Use, 2010). Sensitivity analysis may be performed to test the robustness of the approaches used.

### 2.9 Sample size

Sample sizes for SATs are usually based on powering the study to detect differences from a hypothesised efficacy value in the absence of a comparator arm, while in RCTs they are based on detecting a certain pre-specified effect of treatment *versus* the control (European Medicines Agency, 2020). Likewise, the EC study should be powered to detect a certain effect size between the referent trial and the comparator arm if a confirmatory analysis is planned. In EC studies, the total sample size will be limited by the available trial data such that only the number of EC patients can be varied, and this imposes limits on the achievable statistical power to detect a given effect size. Only marginal benefits may be gained by increasing the size of the external cohort beyond a certain number of patients. Certain design adaptations can increase the number of patients in the EC (e.g., relaxing eligibility criteria or combining comparator treatment strategies), but these approaches can introduce heterogeneity and compromise exchangeability with patients in the referent trial.

Conservativeness should be built into sample size determination for an EC study in order to account for different factors that may reduce the actual sample size available for analysis. For instance, a range of plausible effect sizes should be considered, and an additional percentage added to any calculated sample sizes to account for expected attrition due to the matching or weighting procedures used (e.g., exclusion of unmatched patients) (Rippin et al., 2022). Stricter levels of significance might also be considered in the sample size calculation to account for added variability from combining RW data with SATs or RCTs (e.g., by considering a type I error of 1% instead of 5%). It should be noted that smaller samples may constrain the number of confounders that can be reliably accounted for in the analysis, thereby limiting the exchangeability of the referent trial and ECs and increasing the risk of residual confounding. Very small sample size may also affect the validity of statistical procedures which are only asymptotically valid.

## 3 Conclusion

EC studies are increasingly being used to generate supporting evidence for regulatory and HTA decision making. The application of the well-established TTE framework to the EC setting can improve clarity, transparency, and robustness in the design and analysis of EC studies. This paper has provided commentary on the nuances of applying the TTE framework to EC settings. Further methodological work is needed to provide evidence-based methodological recommendations in applying the TTE framework to EC studies.

## Data Availability

The original contributions presented in the study are included in the article/Supplementary material, further inquiries can be directed to the corresponding author.

## References

[B1] Bakker L. J. Goossens L. M. A. O’Kane M. J. Uyl-de Groot C. A. Redekop W. K. (2021). Analysing electronic health records: the benefits of target trial emulation. Health Policy Technol. 10 (3), 100545. 10.1016/j.hlpt.2021.100545

[B2] Caniglia E. C. Rojas-Saunero L. P. Hilal S. Licher S. Logan R. Stricker B. (2020). Emulating a target trial of statin use and risk of dementia using cohort data. Neurology 95 (10), e1322–e1332. 10.1212/WNL.0000000000010433 32753444 PMC7538212

[B3] Caniglia E. C. Zash R. Jacobson D. L. Diseko M. Mayondi G. Lockman S. (2018). Emulating a target trial of antiretroviral therapy regimens started before conception and risk of adverse birth outcomes. AIDS 32 (1), 113–120. 10.1097/QAD.0000000000001673 29112066 PMC5718935

[B4] Carrigan G. Whipple S. Capra W. B. Taylor M. D. Brown J. S. Lu M. (2020). Using electronic health records to derive control arms for early phase single-arm lung cancer trials: proof-of-concept in randomized controlled trials. Clin. Pharmacol. Ther. 107 (2), 369–377. 10.1002/cpt.1586 31350853 PMC7006884

[B5] Cave A. Kurz X. Arlett P. (2019). Real-world data for regulatory decision making: challenges and possible solutions for europe. Clin. Pharmacol. Ther. 106 (1), 36–39. 10.1002/cpt.1426 30970161 PMC6617710

[B6] Cole S. R. Frangakis C. E. (2009). The consistency statement in causal inference: a definition or an assumption? Epidemiology 20 (1), 3–5. 10.1097/EDE.0b013e31818ef366 19234395

[B7] Committee for Medicinal Products for Human Use (2015). Guideline on adjustment for baseline covariates in clinical trials (EMA/CHMP/295050/2013). European Medicines Agency. London, UK, Available from: https://www.ema.europa.eu/en/adjustment-baseline-covariates-clinical-trials-scientific-guideline.

[B8] Committee for Medicinal Products for Human Use (2010). Guideline on missing data in confirmatory clinical trials (EMA/CPMP/EWP/1776/99 rev. 1). European Medicines Agency. London, UK, Available from: https://www.ema.europa.eu/en/missing-data-confirmatory-clinical-trials-scientific-guideline.

[B9] Cox D. R. (1958). Planning of experiments. Wiley. Hoboken, NJ, USA.

[B10] Curtis L. H. Sola-Morales O. Heidt J. Saunders-Hastings P. Walsh L. Casso D. (2023). Regulatory and HTA considerations for development of real-world data derived external controls. Clin. Pharmacol. Ther. 114 (2), 303–315. 10.1002/cpt.2913 37078264

[B11] Delgado A. Guddati A. K. (2021). Clinical endpoints in oncology - a primer. Am. J. Cancer Res. 11 (4), 1121–1131. 33948349 PMC8085844

[B12] Dong J. Zhang J. L. Zeng S. Li F. (2020). Subgroup balancing propensity score. Stat. Methods Med. Res. 29 (3), 659–676. 10.1177/0962280219870836 31456486

[B13] Emilsson L. Song M. Ludvigsson J. F. (2023). Target trial emulation of aspirin after diagnosis of colorectal polyps. Eur. J. Epidemiol. 38 (10), 1105–1114. 10.1007/s10654-023-01024-1 37322135 PMC10570175

[B14] European Medicines Agency (2020). International Council for harmonisation of technical requirements for registration of Pharmaceuticals for human use (ICH). ICH topic E 9: statistical principles for clinical trials (EMA/CHMP/ICH/436221/2017). European Medicines Agency. Amsterdam, Netherlands, Available from: https://www.ema.europa.eu/en/ich-e9-statistical-principles-clinical-trials-scientific-guideline.

[B15] European Medicines Agency (2023). Real-world evidence framework to support EU regulatory decision-making (EMA/289699/2023). European Medicines Agency. Amsterdam, Netherlands, Available from: https://www.ema.europa.eu/system/files/documents/report/real-world-evidence-framework-support-eu-regulatory-decision-making-report-experience-gained_en.pdf.

[B16] European Medicines Agency Clinical trials in human medicines. Available from: https://www.ema.europa.eu/en/human-regulatory-overview/research-and-development/clinical-trials-human-medicines.

[B17] Fewell Z. Hernán M. A. Wolfe F. Tilling K. Choi H. Sterne J. A. C. (2004). Controlling for time-dependent confounding using marginal structural models. Stata J. 4 (4), 402–420. 10.1177/1536867x0400400403

[B18] Fox M. P. MacLehose R. F. Lash T. L. (2023). SAS and R code for probabilistic quantitative bias analysis for misclassified binary variables and binary unmeasured confounders. Int. J. Epidemiol. 52 (5), 1624–1633. 10.1093/ije/dyad053 37141446 PMC10555728

[B19] Freedland K. E. King A. C. Ambrosius W. T. Mayo-Wilson E. Mohr D. C. Czajkowski S. M. (2019). The selection of comparators for randomized controlled trials of health-related behavioral interventions: recommendations of an NIH expert panel. J. Clin. Epidemiol. 110, 74–81. 10.1016/j.jclinepi.2019.02.011 30826377 PMC6543841

[B20] García-Albéniz X. Hsu J. Hernán M. A. (2017). The value of explicitly emulating a target trial when using real world evidence: an application to colorectal cancer screening. Eur. J. Epidemiol. 32 (6), 495–500. 10.1007/s10654-017-0287-2 28748498 PMC5759953

[B21] Gatto N. M. Vititoe S. E. Rubinstein E. Reynolds R. F. Campbell U. B. (2023). A structured process to identify fit-for-purpose study design and data to generate valid and transparent real-world evidence for regulatory uses. Clin. Pharmacol. Ther. 113 (6), 1235–1239. 10.1002/cpt.2883 36871138

[B22] Greifer N. Stuart E. A. (2021). Choosing the estimand when matching or weighting in observational studies. Available from: http://arxiv.org/abs/2106.10577.

[B23] Hatswell A. J. Deighton K. Snider J. T. Brookhart M. A. Faghmous I. Patel A. R. (2022). Approaches to selecting “time zero” in external control arms with multiple potential entry points: a simulation study of 8 approaches. Med. Decis. Mak. 42 (7), 893–905. 10.1177/0272989X221096070 PMC9459359 35514320

[B24] Hernán M. A. Hernández-Díaz S. (2012). Beyond the intention-to-treat in comparative effectiveness research. Clin. Trials 9 (1), 48–55. 10.1177/1740774511420743 21948059 PMC3731071

[B25] Hernán M. A. Robins J. M. (2006). Estimating causal effects from epidemiological data. J. Epidemiol. Community Health 60 (7), 578–586. 10.1136/jech.2004.029496 16790829 PMC2652882

[B26] Hernán M. A. Robins J. M. (2016). Using big data to emulate a target trial when a randomized trial is not available: table 1. Am. J. Epidemiol. 183 (8), 758–764. 10.1093/aje/kwv254 26994063 PMC4832051

[B27] Hernan M. A. Robins J. M. (2020). Causal inference: what if. Boca Raton, FL, USA: Chapman & Hall/CRC.

[B28] Hernán M. A. Sauer B. C. Hernandez-Diaz S. Platt R. Shrier I. (2016). Specifying a target trial prevents immortal time bias and other self-inflicted injuries in observational analyses. J. Clin. Epidemiol. 79, 70–75. 10.1016/j.jclinepi.2016.04.014 27237061 PMC5124536

[B29] Hornberger B. Rangu S. (2020). Designing inclusion and exclusion criteria, 13. University of Pennsylvania ScholarlyCommons. Philadelphia, PA, USA.

[B30] International Council for Harmonisation of Technical Requirements for Registration of Pharmaceuticals for Human Use (2022). ICH guidelines E8 (R1) on general considerations for clinical studies. European Medicines Agency. Amsterdam, Netherlands, Available from: https://www.ema.europa.eu/en/documents/scientific-guideline/ich-e-8-general-considerations-clinical-trials-step-5_en.pdf.

[B31] Jaksa A. Louder A. Maksymiuk C. Vondeling G. T. Martin L. Gatto N. (2022). A comparison of seven oncology external control arm case studies: critiques from regulatory and health technology assessment agencies. Value Health 25 (12), 1967–1976. 10.1016/j.jval.2022.05.016 35760714

[B32] Kutcher S. A. Brophy J. M. Banack H. R. Kaufman J. S. Samuel M. (2021). Emulating a randomised controlled trial with observational data: an introduction to the target trial framework. Can. J. Cardiol. 37 (9), 1365–1377. 10.1016/j.cjca.2021.05.012 34090982

[B33] Lin D. Y. Psaty B. M. Kronmal R. A. (1998). Assessing the sensitivity of regression results to unmeasured confounders in observational studies. Biometrics 54 (3), 948–963. 10.2307/2533848 9750244

[B34] Lipsitch M. Tchetgen E. T. Cohen T. (2010). Negative controls: a tool for detecting confounding and bias in observational studies. Epidemiology 21 (3), 383–388. 10.1097/EDE.0b013e3181d61eeb 20335814 PMC3053408

[B35] Mansournia M. A. Higgins J. P. T. Sterne J. A. C. Hernán M. A. (2017). Biases in randomized trials: a conversation between trialists and epidemiologists. Epidemiology 28 (1), 54–59. 10.1097/EDE.0000000000000564 27748683 PMC5130591

[B36] Maringe C. Benitez Majano S. Exarchakou A. Smith M. Rachet B. Belot A. (2020). Reflection on modern methods: trial emulation in the presence of immortal-time bias. Assessing the benefit of major surgery for elderly lung cancer patients using observational data. Int. J. Epidemiol. 49 (5), 1719–1729. 10.1093/ije/dyaa057 32386426 PMC7823243

[B37] Martens E. P. Pestman W. R. de Boer A. Belitser S. V. Klungel O. H. (2006). Instrumental variables: application and limitations. Epidemiology 17 (3), 260–267. 10.1097/01.ede.0000215160.88317.cb 16617274

[B38] Murray E. J. Caniglia E. C. Petito L. C. (2021). Causal survival analysis: a guide to estimating intention-to-treat and per-protocol effects from randomized clinical trials with non-adherence. Res. Methods Med. Health Sci. 2 (1), 39–49. 10.1177/2632084320961043

[B39] Nair B. (2019). Clinical trial designs. Indian Dermatology Online J. 10 (2), 193–201. 10.4103/idoj.IDOJ_475_18 PMC6434767 30984604

[B40] National Institute for Health and Care Excellence (2022). NICE real-world evidence framework Guidance. NICE. London, UK, Available from: https://www.nice.org.uk/corporate/ecd9/chapter/overview. 10.57264/cer-2023-0135 PMC10690376 37855246

[B41] Pocock S. J. (1976). The combination of randomized and historical controls in clinical trials. J. Chronic Dis. 29 (3), 175–188. 10.1016/0021-9681(76)90044-8 770493

[B42] Rippin G. (2024). “External comparators and estimands. in Front. Drug Saf. Regul 3. 10.3389/fdsfr.2023.1332040 accepted PMC12443115 40980098

[B43] Rippin G. Ballarini N. Sanz H. Largent J. Quinten C. Pignatti F. (2022). A review of causal inference for external comparator arm studies. Drug Saf. 45 (8), 815–837. 10.1007/s40264-022-01206-y 35895225

[B44] Robins J. M. Hernán M. Á. Brumback B. (2000). Marginal structural models and causal inference in epidemiology. Epidemiology 11 (5), 550–560. 10.1097/00001648-200009000-00011 10955408

[B45] Rodrigues D. Kreif N. Lawrence-Jones A. Barahona M. Mayer E. (2022). Reflection on modern methods: constructing directed acyclic graphs (DAGs) with domain experts for health services research. Int. J. Epidemiol. 51 (4), dyac135. 10.1093/ije/dyac135 PMC9365627 35713577

[B46] Ross M. E. Kreider A. R. Huang Y. S. Matone M. Rubin D. M. Localio A. R. (2015). Propensity score methods for analyzing observational data like randomized experiments: challenges and solutions for rare outcomes and exposures. Am. J. Epidemiol. 181 (12), 989–995. 10.1093/aje/kwu469 25995287

[B47] Rubin D. B. (1974). Estimating causal effects of treatments in randomized and nonrandomized studies. J. Educ. Psychol. 66 (5), 688–701. 10.1037/h0037350

[B48] Seaman S. R. White I. R. (2013). Review of inverse probability weighting for dealing with missing data. Stat. Methods Med. Res. 22 (3), 278–295. 10.1177/0962280210395740 21220355

[B49] Seo S. K. (2023). External control arms: we’re not on cruise control yet. Clin. Pharmacol. Ther. 114 (2), 249–251. 10.1002/cpt.2963 37475671

[B50] Sola-Morales O. Curtis L. H. Heidt J. Walsh L. Casso D. Oliveria S. (2023). Effectively leveraging RWD for external controls: a systematic literature review of regulatory and HTA decisions. Clin. Pharmacol. Ther. 114 (2), 325–355. 10.1002/cpt.2914 37079433

[B51] Toh S. Hernández-Díaz S. Logan R. Robins J. M. Hernán M. A. (2010). Estimating absolute risks in the presence of nonadherence: an application to a follow-up study with baseline randomization. Epidemiology 21 (4), 528–539. 10.1097/EDE.0b013e3181df1b69 20526200 PMC3315056

[B52] U.S. Food and Drug Administration (2018). Framework for FDA’s real-world evidence program. Silver Spring, MD, USA. U.S. Food & Drug Administration. Available from: https://www.fda.gov/media/120060/download.

[B53] U.S. Food and Drug Administration (2023). Considerations for the design and conduct of externally controlled trials for drug and biological products: guidance for industry [DRAFT]. U.S. Department of Health and Human Services. Silver Spring, MD, USA, Available from: https://www.fda.gov/regulatory-information/search-fda-guidance-documents/considerations-design-and-conduct-externally-controlled-trials-drug-and-biological-products.

[B54] Willems S. J. W. Schat A. van Noorden M. S. Fiocco M. (2018). Correcting for dependent censoring in routine outcome monitoring data by applying the inverse probability censoring weighted estimator. Stat. Methods Med. Res. 27 (2), 323–335. 10.1177/0962280216628900 26988930

[B55] Young J. G. Stensrud M. J. Tchetgen Tchetgen E. J. Hernán M. A. (2020). A causal framework for classical statistical estimands in failure-time settings with competing events. Statistics Med. 39 (8), 1199–1236. 10.1002/sim.8471 PMC7811594 31985089

[B56] Zhu Y. Hubbard R. A. Chubak J. Roy J. Mitra N. (2021). Core concepts in Pharmacoepidemiology: violations of the positivity assumption in the causal analysis of observational data: consequences and statistical approaches. Pharmacoepidemiol Drug Saf. 30 (11), 1471–1485. 10.1002/pds.5338 34375473 PMC8492528

